# Insights into the Mechanism of Ligand Binding to Octopine Dehydrogenase from *Pecten maximus* by NMR and Crystallography

**DOI:** 10.1371/journal.pone.0012312

**Published:** 2010-08-19

**Authors:** Sander H. J. Smits, Tatu Meyer, Andre Mueller, Nadine van Os, Matthias Stoldt, Dieter Willbold, Lutz Schmitt, Manfred K. Grieshaber

**Affiliations:** 1 Institute of Biochemistry, Heinrich-Heine-University, Duesseldorf, Germany; 2 Institute of Zoophysiology, Heinrich-Heine-University, Duesseldorf, Germany; 3 Institute of Physical Biology, Heinrich-Heine-University, Duesseldorf, Germany; 4 Institute of Structural Biology and Biophysics (ISB-3), Research Centre Jülich, Jülich, Germany; Massachusetts Institute of Technology, United States of America

## Abstract

Octopine dehydrogenase (OcDH) from the adductor muscle of the great scallop, *Pecten maximus*, catalyzes the NADH dependent, reductive condensation of L-arginine and pyruvate to octopine, NAD^+^, and water during escape swimming and/or subsequent recovery. The structure of OcDH was recently solved and a reaction mechanism was proposed which implied an ordered binding of NADH, L-arginine and finally pyruvate. Here, the order of substrate binding as well as the underlying conformational changes were investigated by NMR confirming the model derived from the crystal structures. Furthermore, the crystal structure of the OcDH/NADH/agmatine complex was determined which suggests a key role of the side chain of L-arginine in protein cataylsis. Thus, the order of substrate binding to OcDH as well as the molecular signals involved in octopine formation can now be described in molecular detail.

## Introduction

Several molluscan species, in particular cephalopods are known for their vivid swimming performances. Also among the usually slow moving or even sedentary bivalves, active species are known such as the Pectinid scallops which exhibit jumping and swimming movements when attacked by predatory starfish [1]. The valve clapping movements shown during escape response are powered by a single valve adductor muscle, the scallop, which derives ATP for this strenuous muscle activity mainly from trans-phosphorylation of phospho-L-arginine and to a lesser extent by anaerobic glycolysis. Already during escape response or subsequent recovery the animals restore the phospho-L-arginine pool using anaerobic glycolysis which is terminated by the formation of octopine, a reaction catalyzed by the octopine dehydrogenase [2]. This enzyme received considerable interest in the past, mainly because of its analogy to lactate dehydrogenase (LDH). However, the reaction mechanism seems to be different in both enzymes.

**Figure 1:**
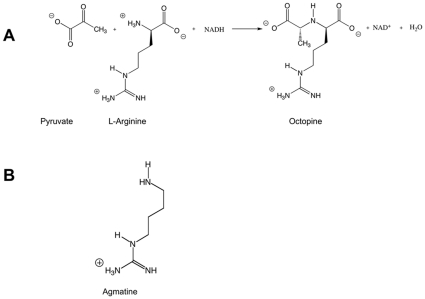
Chemical structures A) Reaction scheme for the reductive condensation of pruvate and L-arginine mediated by OcDH. B) Chemical structure of agmatine.

Octopine dehydrogenase (OcDH, N^2^-(D-1-carboxyethyl)-L-arginine:NAD^+^-oxidoreductase, EC 1.5.1.11) catalyzes the reductive condensation of L-arginine and pyruvate in the presence of NADH to yield D-octopine [(*R*)-N^2^-(1-carboxyethyl)-L-arginine], NAD^+^, and water (Figure 1). This enzyme was first discovered in 1959 by van Thoai and Robin in extracts from the adductor muscle of the great scallop, *Pecten maximus (P. maximus)* [3]. It has also been found in muscle tissue of many other marine as well as in some limnic invertebrates [4].

OcDH synthesizes octopine during escape or when recovering from exhaustive, anaerobic muscle work. Octopine can, therefore, be considered like lactate as an end product of anaerobic glycolysis in many animals [4].

During the last 50 years, the enzymatic characteristics of OcDH from the adductor muscle of *P. maximus* have been extensively studied and numerous basic informations have been published [5], [6], [7], [8]. Recently, cloning and heterologous expression of OcDH from *P. maximus* were accomplished [9] and the crystal structures of OcDH in complex with NADH, NADH/L-arginine as well as NADH/pyruvate were reported [10]. The biochemical characterization underlined the substrate specificity of OcDH since next to L-arginine only L-cysteine and L-alanine can be used as a substrate for OcDH from the pool of all natural amino acid tested. However, the activity for the latter two amino acids is severely reduced (1.2% for L-cysteine and 0.3% for L-alanine, respectively) [10].

Despite the physiological, biochemical and structural information available for OcDH from *P. maximus*, controversial discussions about the kinetic reaction mechanism remain. Deduced from kinetic analyses, two mechanisms for the OcDH-catalyzed reaction have been proposed. Baici *et al* (1974) proposed, based on kinetic and fluorometric data, that the apo enzyme forms a binary complex in the presence of NADH to which the other two substrates bind in a compulsory ordered sequential mechanism [11]. This step should result in the formation of a Schiff base. The subsequent hydride transfer from NADH to the Schiff base reduces this transient intermediate to D-octopine. In contrast, Schrimsher and Taylor (1984) described a random-ordered mechanism. After the formation of the binary OcDH/NADH complex, L-arginine and pyruvate are supposed to bind randomly [12]. Thus, it is still uncertain up to now in which order the substrates bind to the enzyme before the product D-octopine is formed.

Recently, the crystal structure of OcDH from *Pecten maximus* was solved [10]. The enzyme consists of two domains, an NADH binding domain and a so-called octopine-binding domain. The latter one forms a helix-kink-helix motif located at the L-arginine binding site. Furthermore, a catalytic dyad comprising a His–Asp proton relay system and an additional Arg-sensor (Arg324) were identified. From these crystal structures, a sequential order of substrate binding was postulated. NADH is bound to the Rossmann fold motif localized in domain I. This triggers an interaction of Arg324 (domain II) with the pyrophosphate moiety of NADH. Thereby a stable conformation of OcDH is secured which allows L-arginine to bind. As last substrate pyruvate binds and is immediatle condensated with L-arginine resulting in octopine formation.

We like to stress that in the crystal, domain I and domain II are stabilized in the presence of NADH by two different sets of interactions. First, Arg324 located in domain II binds the pyrophosphate moiety of NADH bound to domain I thereby acting as a “NADH-sensor”. This already closes the protein. Second, the His-tag located in the cleft of both domains clues together these two parts. Through this sort of artificial arrangement, the pyruvate binding site is generated that would not be present in solution [10], This allowed us to solve the structure of OcDH/NADH-pyruvate which in solution is not observed.

Thus the formation of the OcDH/NADH complex should precede the binding of L-arginine, which then induces a rotational movement (via the helix-kink-helix motif) of the two domains towards each other. This movement coordinates the placement of the α-ketogroup of pyruvate and the α-amino group of L-arginine in close proximity to each other resulting in the subsequent formation of a Schiff base which is immediately reduced to D-octopine and thus prevents the reduction of pyruvate to lactate. The formation of the latter has never been observed in *P. maximus*, which indicates that pyruvate binding to OcDH does not occur spontaneously.

Such a conformational change has also been proposed for the Saccharopine Dehydrogenase from *Saccharomyces cerevisiae*, which catalyzes the reversible pyridine nucleotide-dependent oxidative deamination of saccharopine to yield L-lysine and α-ketoglutarate (α-KG) [13]. This reaction, in principle, corresponds to the backward reaction of OcDH. Here, the binding of saccharopine is suggested to induce a conformational change, which brings the two domains closer together, narrowing the active site cleft. This positions the cofactor NADH and the substrate in close proximity allowing hydride transfer by NADH [13].

In solution, however, no activity can be observed when pyruvate is added as a substrate to OcDH in the presence of NADH. Such a reaction could possibly generate lactate in analogy to lactate dehydrogenase. However, the crystal structures of OcDH revealed that the conformational change observed by L-arginine binding is a prerequisite to form the pyruvate binding site [14]. Despite the new information derived from the crystal structures of OcDH, the following additional questions still remain enigmatic: (I) Is the order of formation of a ternary OcDH/NADH/L-arginine complex inducing conformational changes in aqueous solution? (II) Is the side-chain of L-arginine dictating these conformational changes?

Here, we have applied two different methods, nuclear magnetic resonance (NMR) and X-ray crystallography to address these questions.

## Materials and Methods

All chemicals used were of analytical grade and were used without further purification. NADH and NAD^+^ were obtained from Roche, pyruvate L-arginine and agmatine from Sigma.

Cloning of the OcDH gene, heterologous, large scale expression and determination of enzymatic activity of OcDH, were performed as described previously (Mueller et al, 2007; Smits et al, 2008b).

### Expression and Purification

Expression and purification of isotope labeled OcDH-His_5_: For the expression of U-[^15^N] labeled OcDH M9 Minimal media (0.6% (w/v) Na_2_HPO_4_, 0.3% (w/v) KH_2_PO_4_, 0,05% (w/v) NaCl, 0.4% (w/v) Glucose, 1 mM MgSO_4_, 0.3 mM CaCl_2_, 1 µg mL^−1^ Biotin, 1 µg mL^−1^ Thiamin, 50 µg mL^−1^ EDTA 8,3 µg mL^−1^ FeCl_3_, 0.84 µg mL^−1^ ZnCl_2_, 0.13 µg mL^−1^ CuCl_2_, 0.1 µg mL^−1^ CoCl_2_, 0.1 µg mL^−1^ H_3_BO_3_, 16 ng mL^−1^ MnCl_2_, pH 7.0) supplemented with 0.1% (w/v) ^15^N-ammoniumchlorid as only nitrogen source was used. Five liter minimal media were inoculated with an over night culture and cells were grown for 12 h at 37°C. Cells were then induced by the addition of 0.35 mM IPTG and the expression temperature was lowered to 18°C for another 36 hours. Purification was performed as described before [9] and finally OcDH was dialysed against 50 mM Na-phosphate buffer pH 6.8, 1 mM DTT and 1 mM EDTA.

### NMR experiments

Expression and purification of U-[^15^N] labeled OcDH was carried out as described above. NMR samples for titration experiments contained 0.25 mM U- [^15^N] labeled OcDH in aqueous solution (50 mM potassium phosphate, pH 6.8, 1 mM EDTA, 1 mM DTT and 6% (v/v) 2D_2_O). All NMR experiments were carried out at 298 K on a Varian UnityINOVA spectrometer, equipped with a 5 mm cryogenic Z-axis PFG-1H triple resonance probe at a proton frequency of 800 MHz. For each concentration of ligands a 2D TROSY-type [15] ^1^H-^15^N correlation experiment was recorded with 69 ms maximum 15N evolution time and 16 accumulated scans t1 per time increment. The concentration of NADH was increased from 75 µM to 3 mM in 6 steps, NAD^+^ from 75 µM to 5 mM in 7 steps and L-arginine from 75 µM to 8 mM in 6 steps with 3 mM NADH present in all steps.

Spectra were processed using the Varian VNMRJ 1.1D software and analyzed with the program CARA [15]. For determination of dissociation constants the incremental shift of individual resonance peaks were extracted as vector lengths from each series of titration spectra and subsequently plotted against ligand concentration. Curves were fitted to following equation (one-site saturation model):

Δδ  =  (Δδmax · [L])/(Kd + [L]).

Here, Kd is the dissociation constant, [L] the ligand concentration, Δδ is the change of the chemical shift, Δδmax the maximal change of the chemical shift of an individual resonance.

### X-ray crystallography and structure determination

As it was not possible to obtain crystals of the OcDH/NADH-agmitine complex we used soaking to obtain the agmatine bound OcDH structure. This means that OcDH protein crystals were transferred into a solution containing agmatine, which diffuses into the crystal and is specifically bound. Therefor crystals of OcDH-His_5_ were grown as described previously [10]. In brief, crystals grew by mixing protein solution (20 mg mL^−1^) with reservoir solution, (100 mM MES pH 6.0–7.0) and Na-citrate ranging from 1.0 to 1.2 M, in a 1∶1 ratio. Crystals appeared after approximately 5 days. Prior to crystallization, 0.8 mM NADH was added to the protein solution. Agmatine-bound crystals were obtained by soaking NADH-bound OcDH crystals in 100 mM MES pH 7.0, 1.15 M Na-citrate, 0.8 mM NADH containing 20 mM agmatine for at least 8 hours. Suitable crystals were washed in 100 mM MES pH 7.0, 30% (v/v) ethylene glycol, 1.15 M Na-citrate and directly frozen in liquid nitrogen.

### Data collection and structure determination

A dataset of OcDH/NADH/agmatine at 2.8 Å was collected at beamline BW7A, EMBL, DESY, Hamburg (Germany). Detailed information on data collection statistics are shown in Table 1. The dataset was processed with the XDS program package [16] and phased and refined by rigid body refinement using the OcDH/NADH/L-arginine structure as template (PDB code: 3C7C) and further refined using REFMAC5 [17] and COOT [18]. Dataset and refinement statistics are summarized in Table 1. The final OcDH/NADH-Agmatine structure was compared with the OcDH/NADH complex by superimposing both structures. Here domain I (NADH binding domain) was used as ankerpoint.

**Table 1:** Refinement statistics of the ODH/NADH-agmatine complex.

Crystal parameters at 100 K	Agmatine (NADH)
Space group	P4_1_2_1_2
Unit Cell parameters	
a = b, c (Å)	96.2,96.2 117.4
α, = β = γ (deg.)	90.0
Wavelength (Å)	0.8148
Resolution (Å)	20–2.8 (3.0–2.8)
Mean redundancy	4.4 (4.5)
Unique reflections	11968 (2282)
Completeness (%)	90.0 (92.5)
I/σ	13.6 (4.5)
R_syme_^a^	10.3 (36.3)
***Refinement***	
R-factor (%)	21.1 (32.2)
Rfreec (%)	26.5 (36.3)
rmsd from ideal	
Bond lengths (Å)	0.09
Bond angles (deg.)	1.23
Average B-factors (Å^2^)	28.48
**Ramachandran plot**	
Most favored (%)	98.2 (396 residues)
Allowed (%)	1.8 (7 residues)
Generously allowed (%)	0
Disallowed (%)	0
***Model content***	
Monomers/ASU	1
Protein residues (start methionine is not visible)	2–404
Ligand	1 NADH, 1 Agmatine

Refinement statistics were obtained from REFMAC5 [17] and Ramachandran analysis was performed using PROCHECK [28]. ^a^Rsym is defined as 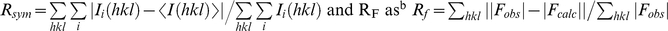. R_free_ is calculated as R_F_ but for 5% randomly chosen reflections that were omitted from all refinement steps.

### Figure preparation

Structure figures were prepared using PyMol (http://pymol.sourceforge.net/).

## Results

### Substrate induced chemical shift changes observed by NMR spectroscopy

Solution NMR spectroscopy was applied as an independent approach to verify the results from X-ray crystallography [10]. An advantage of solution NMR compared to X-ray crystallography is that all measurements are performed in aqueous solution. No crystals are required to investigate substrate binding. Furthermore, NMR not only provides insights into the thermodynamics of ligand binding, but it is also well suited to detect structural rearrangements upon ligand binding. Therefore, a series of protein heteronuclear single quantum coherence (HSQC) spectra were recorded with increasing concentrations of ligand in the sample, also called “HSQC titration” [19]. If a full sequence specific assignment of the HSQC resonances is present and a three-dimensional solution structure of the protein, HSQC titrations allow mapping of the ligand-binding site onto the protein surface and provide insights into the thermodynamics of ligand binding, possibly the mode of binding, and in favorable cases even the dissociation constant of the protein–ligand complex [19], [20].

To perform HSQC-titrations, OcDH was uniformly isotope labeled by using ^15^N-ammonium chloride as the sole nitrogen source during heterologous expression of OcDH in *E. coli*. ^15^N-labeled OcDH was then used to record 2D ^1^H-^15^N-TROSY-HSQC spectra of the enzyme. The resolution and spatial dispersion of the ^1^H-^15^N cross peaks were excellent, 367 separated backbone ^1^H-^15^N cross peaks could be detected, which was well in accordance with the expected number of resonances for OcDH (399 residues). We used this spectrum as a reference for the detection of chemical shift perturbations caused by binding of NADH, L-arginine and/or pyruvate.

Unfortunately, we were not able to establish full assignments due to poor deuterium to proton back-exchange of the per-deuterated and ^13^C-^15^N-labelled OcDH NMR sample. About 25% of the backbone amides did not exchange with H_2_O even under denaturing conditions. Here, suitable refolding procedures for OcDH after full deuterium to proton back-exchange under denaturating conditions could not be established.

**Figure 2:**
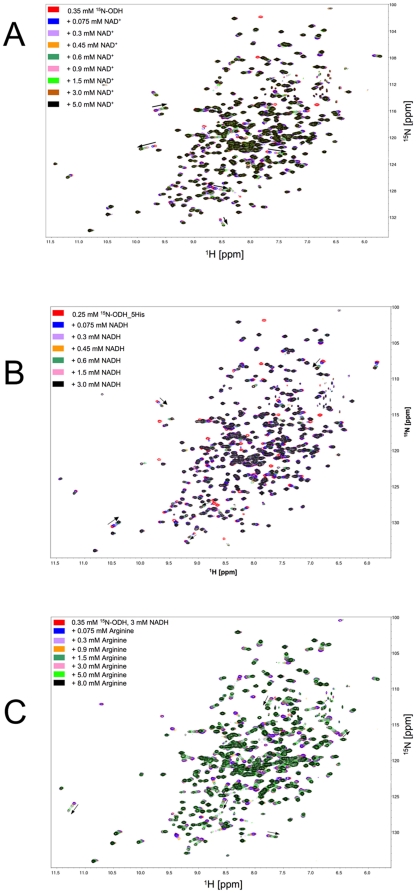
Superposition of (^1^H-^15^N) TROSY-HSQC-spectra of OcDH-His_5_ after titration with different substrate concentrations. The spectra were collected at 296 K at a proton resonance of 800 MHz. The initial protein concentration was 0.25 mM. A) NAD^+^ titration. The NAD^+^ concentration was raised in seven steps from 0.075 mM (blue line) to 5 mM (black line). B) NADH titration. The NADH concentration was raised in six steps from 0.075 mM (blue line) to 3 mM (black line) C) L-arginine titration. NADH was added prior to titration at a concentration of 3 mM. The L-arginine concentration was raised from from 0.075 mM (blue line) to 8 mM (black line). Black arrows indicate the resonances which movements were used for the calculation of the dissocation constant.

**Figure 3:**
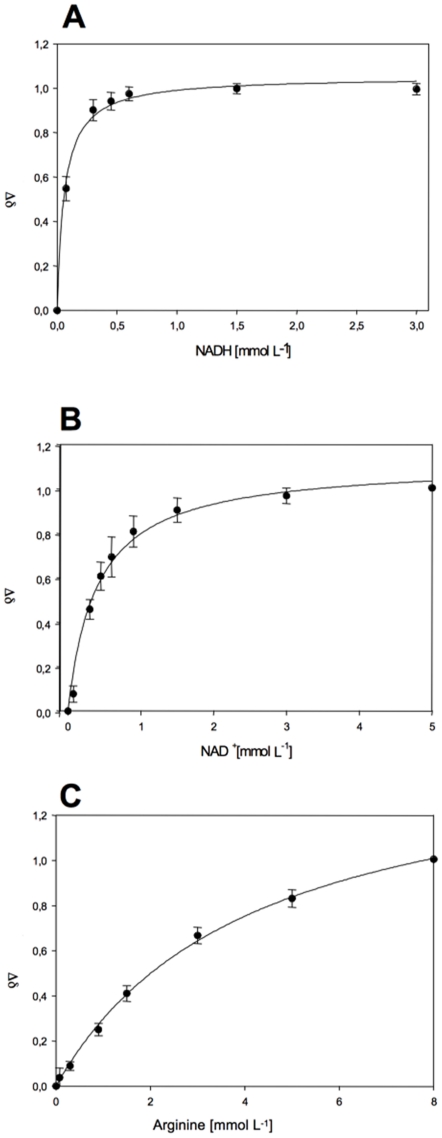
Determination of the dissociation constant of NADH (A), NAD^+^ (B) and L-arginine (C). Plotted are the normalised chemical shifts of specific amid resonances at different substrate concentrations.

Nevertheless, for binding studies, NADH was first titrated to ^15^N-labeled OcDH with increasing concentrations of NADH ranging from 0.075 mM to 3 mM (Figure 2a) and 2D-TROSY-HSQC spectra were recorded at each concentration. Superposition of the ^15^N-^1^H-TROSY spectra of the apo-enzyme and the OcDH/NADH complex revealed that several peaks shifted to different resonance frequencies, which were directly correlated to the NADH concentrations. The measured differences of the chemical shifts of at least five randomly selected peaks were used for the calculation of dissociation constant (K_d_) for NADH (Figure 3). Applying a single binding site fit a *K_D_*  = 0.062±0.007 mM for NADH was determined. For NAD^+^ a *K_D_* of 0.4±0.05 mM was obtained (Figure 2b and 3b). In the absence of NADH superposition of ^1^H-^15^N-spectra recorded after titration of L-arginine as well as pyruvate to the [^15^N]-OcDH solution did not yield chemical shift perturbations of cross peaks (data not shown). This suggests that NADH is the first substrate to bind to OcDH.

Since no shifts of amide cross peaks had been observed with either L-arginine or with pyruvate in the absence of NADH, OcDH was saturated with 3 mM NADH prior to titration of one of these substrates. Further addition of pyruvate up to 10 mM did not result in any shift of amide cross peaks (data not shown). However, when the OcDH/NADH complex was titrated with L-arginine in concentrations ranging from 0.075 mM up to 8 mM, superposition of the spectra revealed a large number of resonance peaks being shifted. The dependence of the respective chemical shift differences of the L-arginine enabled us to calculate a *K_D_* value of 4.1±0.31 mM for the binding of L-arginine to the binary OcDH/NADH complex (Figure 1c and 2c). These values obtained by NMR (Table 2) are in good agreement with reported *K_d_*'s [11]. Furthermore, it strongly suggests that L-arginine is the second substrate to bind to OcDH as postulated recently [10].

**Table 2:** Dissociation constants of substrate binding to octopine dehydrogenase.

	NMR	Literature (11)
titration experiment	K_d_	K_d_
	(mM)	(mM)
NADH in OcDH	0.062±0.007	0.020
NAD^+^ in OcDH	0.4±0.05	0.38
L-arginine in OcDH + NADH	4.1±0.31	
Pyruvate in OcDH + NADH	N.b.d	

The dissociation constants for NADH, NAD^+^, L-arginine as determined by NMR as well as the values derived from the literature are reported. “N.b.d”: no binding detectable under the experimental set-up.

In addition, chemical shift changes in general indicate different chemical environment of the respective nuclei. The chemical environment can change due to direct interaction with the substrate or due to substrate binding induced conformational changes, even minor ones. The sheer number of chemical shift changes in the finger print region upon L-arginine binding indicates that much more residues are affected upon substrate binding as could be explained by a sole and physical interaction of the protein with the substrate. Thus, L-arginine binding obviously induced conformational changes in OcDH. **What induces the conformational change of OcDH upon L-arginine binding?**

**Figure 4:**
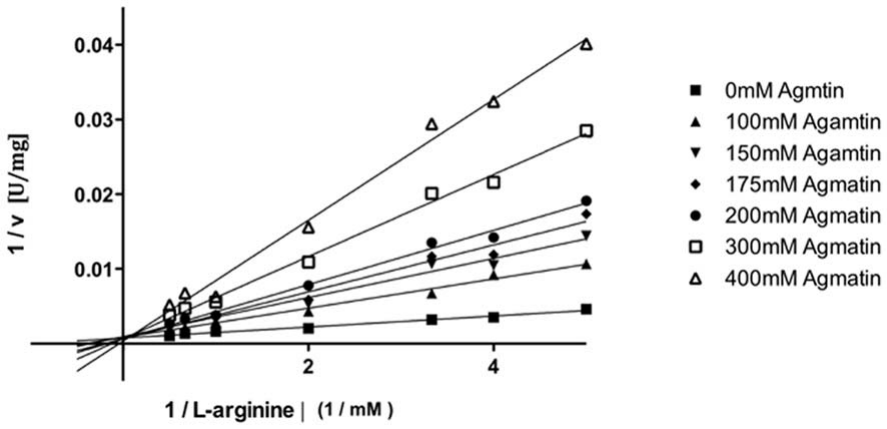
Inhibition study of OcDH by using agmatine. A Lineweaver-Burk plot is shown for the activity of OcDH at fixed L-arginine concentration and increasing levels of agmatine. The double reciprocal representation shows that all concentration of the inhibitor analysed crossed the y-axis at the same point, which indicates the OcDH is inhibited by agmatine in a competitive manner.

Agmatine is the decarboxylation product of arginine (see the chemical structures in Figure 1). This molecule obviously opens the possibility to dissect the individual influence of the α-carboxylate moiety and the side chain of L-arginine. Using agmatine in activity assays instead of L-arginine revealed no activity. Two possible reasons can explain this lack of activity. Either agmatine is not binding or the carboxyl group, which is present in L-arginine but not in agmatine, is crucial for the condensation reaction. To distinguish between the two scenarios, inhibition studies were performed (Figure 4). The evaluation of the data clearly demonstrates that agmatine acts as a competitive inhibitor with a K_i_ of 40±2 mM. This implicates that agmatine and L-arginine bind to the same binding site in OcDH.

Consequently, we were wondering if the binding of agmatine is inducing a similar conformational change in OcDH as L-arginine as observed in NMR (reported here) and X-ray studies [10]. Since no sequence assignment could be established in the NMR experiments, we solved the structure of the OcDH/NADH-Agmatine complex by X-ray crystallography. This structure should give an answer whether or not a domain closure is induced by the side chain of agmatine or by the functional important coordination of the carboxyl group of L-arginine by His212. A 2.8 Å dataset was collected and initial phases were obtained by a rigid body refinement with the OcDH/NADH-pyruvate structure as template. The structure was further built using COOT [18] and several rounds of refinement using REFMAC5 [17]. Dataset statistics and refinement statistics as well as final model contents are summarized in Table 1.

**Figure 5:**
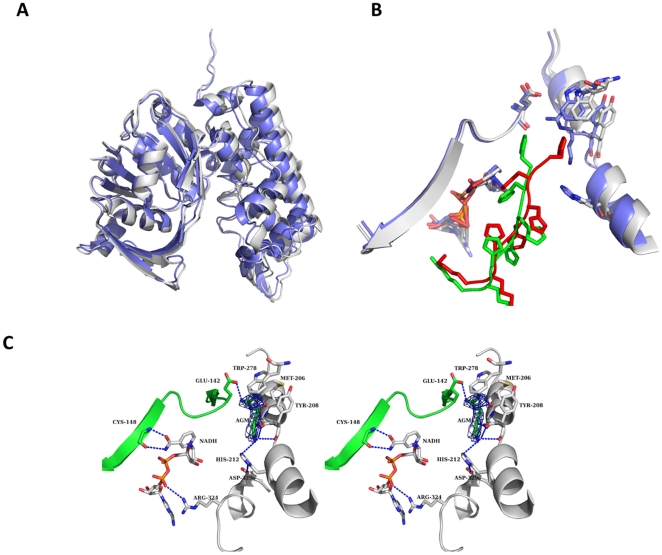
Structural comparison A) Superposition of the OcDH/NADH (white) and the OcDH/NADH-agmatine (blue) complexes highlight the inward rotation of domain II. B) Superposition of the agmatine binding site. Shown is the binding site in the OcDH/NADH complex (white) and from the OcDH/NADH-agmatine complex (blue).the reorientation of the his_5_-tag is shown by the representation of both his_5_-tag (red for the OcDH/NADH complex and green for OcDH/NADH/-agmatine complex) C) Stereoview of the agmatine-binding pocket of OcDH. The agmatine-binding pocket is located in domain II, directly at the N-terminal helix-kink-helix motif of domain II. Agmatine is coordinated by the side chain Trp278, backbone interactions with Tyr208 (domain II) and Glu142 (domain I). NADH is bound in a canonical fashion to the Rossman fold, while interactions of the amide side chain of the nicotine amide ring with the backbone atoms of Cys148 (domain I) ensure the syn-conformation. The electron density of an omit map contoured at 1 σ for agmatine is shown as blue mesh.

The overall structure of the complex is provided in Figure 5a. The structure of OcDH/NADH-agmatine appears more closed than the OcDH/NADH structure (Figure 5a). Here domain I, the NADH binding domain, was used as an ankerpoint as no structural rearrangements where observed inside this domain. As described above the his_5_-tag of OcDH is crucial for crystallization [14]. It is protruding into the cleft of both domains thereby locking OcDH in a conformation enabling crystal contact formation. In the OcDH/NADH-agmatine complex the his_5_-tag is positioned differently as in the OcDH/NADH complex (Figure 5b). This conformational change is only observed when the agmatine soak into the OcDH/NADH complex crystals was successful and agmatine was bound to OcDH. This indicates that the reorientation is a consequence of agmatine binding to the OcDH/NADH crystals.

Already during the initial round of refinement after rigid body refinement and manual building it became obvious that agmatine is bound inside the structure in a fashion similar to L-arginine in the OcDH/NADH-L-arginine complex (Figure 5C). The guanidinium headgroup of agmatine is coordinated via interactions with the side chains of Glu142 (domain I) and Trp278 (domain II) as well as backbone interactions with Met206 (domain II). The Cβ atom is bound via backbone interactions of Ser207. The density of agmatine is of reasonable quality. However, at a comparable resolution to the OcDH/NADH-L-arginine complex, the density is not of the same quality than the density of L-arginine. This suggests that agmatine is bound inside the crystal less well than L-arginine.

However, the binding of agmatine induces a similar rotational movement of domain II towards domain I (Figure 5a), which is almost identical to the domain closure observed in the OcDH/NADH-L-arginine structure [10]. This is reflected in the RMSD value between the L-arginine and agmatine structure is 0.6 Å over 399 Cα atoms whereas the comparison with OcDH/NADH complex is 1.5 Å over 399 Cα atoms.

In both structures (agmatine and L-arginine complex) Arg324 forms a salt bridge with the pyrophosphate moiety of NADH. As a consequence of the domain movement, the distance of this salt bridge is reduced from 4.3 Å (NADH complex) to 3.8 Å (L-arginine and agmatine complex), which of course will strengthen this interaction.

As stated above, the conformational change observed here as well as the reorientation of the his_5_-tag was never detected in the crystals of the OcDH/NADH complex. Furthermore, data sets of unsuccessful soaking of agmatine into the crystals revealed structures identical to the OcDH/NADH complex with no reorientation of the his_5_-tag or domain closure and these data sets had no significant change in the unit cell axis. This suggests, that a significant reorientation of the two domains occurs during the process of binding of agmatine into the crystals. Obviously the conformational change is tolerated by the crystal lattice and not dictated by packing forces. Further support for this interpretation comes from a closer inspection of the his_5_-tag that protrudes in both structures (OcDH/NADH and OcDH/NADH-agmatine complex) in the cleft between both domains. In the OcDH/NADH complex water molecules establish a network of interactions between the protein and the his_5_-tag [14]. In contrast direct interactions between the his_5_-tag and OcDH are observed in the OcDH/NADH-agmatine complex similar to the ones observed in the OcDH/NADH-L-arginine complex. This demonstrates that the substrate induced conformational change in the protein generates a new set of interactions that are only possible in the ternary complex. Due to this rearrangement, the diffraction quality of these crystals decreased due to the ligand-induced rotation, which is reflected in a lower resolution (2.1 Å for the OcDH/NADH complex and 2.8 Å for OcDH/NADH-agmatine complex, respectively).

## Discussion

Several studies have been undertaken to gain insights into the kinetic mechanism of the reductive condensation of octopine and its reversible oxidation. The results of spectrometric and fluorometric studies indicated that NADH binds initially to the apo enzyme, followed by L-arginine forming the OcDH/NADH-L-arginine complex [21]. These results questioned the results of Schrimsher and Taylor who proposed, based on kinetic and inhibitor studies, that NADH binds first followed by a random binding of L-arginine and pyruvate [12]. Based on structural evidence a binding sequence of NADH, L-arginine and pyruvate leading to the active complex was suggested [10]. In order to clarify these discrepancies and also to answer the question how the reduction of pyruvate to lactate instead of the formation of octopine is prevented, we gained additional information on the substrate binding mechanism employing solution NMR and X-ray crystallography.

The NMR titrations demonstrated that neither addition of L-arginine nor of pyruvate to the apo enzyme did induce any differences in amide cross peaks in the absence of NADH. Obviously, NADH respectively NAD^+^ must bind first to OcDH which has already been shown for some other dehydrogenases [22]. Indeed, L-arginine binds to OcDH when pre-saturated with NADH as demonstrated by the shift of amide cross peaks in the corresponding NMR experiments. The dissociations constants for NADH and NAD^+^ are in good agreement with the binding constants reported for octopine formation by Doublet and Olomucki who used fluorometric studies obtaining values of 0.02 mM and 0.38 mM for NADH and NAD^+^ respectively [8]. The dissociation constant for L-arginine is not reported in literature so far. However during the recovery of *P. maximus* the L-arginine concentrations are in the mM range in this organism. Therefore the measured K_d_ of 4.1±0.3 mM for L-arginine to OcDH is in accordance with the *in vivo* levels of L-arginine [4]. This demonstrates that L-arginine is capable of binding to the binary OcDH/NADH complex. However, no shifts of amide cross peaks were observed when pyruvate was added to a NADH-saturated enzyme. This is in agreement with biochemical data of OcDH were no activity is observed when pyruvate is added to OcDH/NADH [9], [10].

The results of the NMR-spectroscopic investigations not only suggest a clear order and seuqnece of substrate binding, but also show that L-arginine binding is associated with a conformational change in solution. This confirms the conformational change substantiated in the X-ray structure of the substrate bound complex [10].

NADH binding introduces a small conformational change, as it is known for most dehydrogenases [23]. Only a few amide cross peaks shift in the NMR spectra and the crystal structure evidently shows that only a slight conformational change is required to stabilize the orientation of both domains towards each other via the interaction of Arg324 with NADH, thereby generating the binding site for the second substrate, L-arginine as seen in the published crystal structure [10].

Pyruvate binding to the OcDH/NADH binary complex could not be detected through a shift of amide cross peaks in the NMR experiments. The binding site of pyruvate is in proximity to the NADH allowing hydride transfer. This however would lead to the formation of lactate, which cannot be detected *in vivo* as well as *in vitro* experiments. As mentioned before, this leads to the conclusion that L-arginine binds prior to pyruvate and is the second substrate that binds in a sequentially ordered mechanism. L-arginine binding is associated with a conformational change, which generates the binding site for pyruvate [10] and allows pyruvate to be located in close proximity to NADH. A similar mechanism was described for the bacterial *N*-(1-D-carboxylethyl)-L-norvaline dehydrogenase (CENDH) from *Athrobacter spec*. by Britton *et al*. [24]. In the CENDH mechanism the amino-acid substrate is reported to bind to the enzyme/coenzyme complex before pyruvate. In the above proposed sequential ordered mechanism, the reduction of pyruvate to lactate without the need for L-arginine is avoided, which is in line with the published data were no lactate could be detected albeit in *Pecten jacobaeus* [25].

Since we were not able to perform a sequence specific assignment of the amide cross peaks of OcDH via NMR we investigated the conformational change further by an inhibitor, namely agmatine, which is a L-arginine analog lacking the carboxyl group. We crystallized and solved the structure of the OcDH/NADH-agmatine complex. The his_5_-tag as well as the coenzyme NADH has been essential for the crystalisation of OcDH [14]. However the his_5_-tag binds in between both domains and is localized near the L-arginine as well as the pyruvate binding site. By soaking of the substrates into preformed crystals the structures of both OcDH/NADH-substrate complexes were solved and the binding sites revealed. Not only the binding sites were verified by an extensive mutational analysis [10], but now the conformational changes were also observed in solution by NMR spectroscopy. OcDH-His_5_ in solution never appeared to form a dimeric or higher oligomeric species, as analysed by size exclusion chromatography or native gel electrophoresis (data not shown). Furthermore a his-tag induced oligomerisation and a subsequent conformational change would have been detected in the solution NMR experiments. In all cases (apo enzyme, NADH and substrates) the T2 relaxation rate did not change. The determined T2 relaxation rate is in agreement with a monomeric OcDH under the condition of the experiments, indicating that even at the concentrations required for the NMR experiments OcDH remains monomeric (data not shown). This suggests that the conformational change observed is not induced by the his_5_-tag but rather via the addition of the inhibitor agmatine.

Here a similar conformational change was observed as in the recently reported OcDH/NADH-L-arginine complex. This implies that the carboxyl group of the L-arginine amino acid is not responsible for the domain closure. OcDH has been shown to be specific for L-arginine and any other amino acid reduces the activity of OcDH by almost a factor of 100 [10]. Thus, the binding of the side chain of L-arginine via interaction with both domains of the protein is likely crucial for triggering conformational change required to generate the bindings sites of the other substrates.

In the structure of the saccharopine dehydrogenase from *Saccharomyces cerevisiae* the ternary complex model does not represent the catalytically competent conformation of the enzyme as the substrate and cofactor are too far apart for catalysis to occur (5.7 Å) [13]. This suggests that a similar conformational change as seen in OcDH is required during the reaction cycle. Besides, the saccharopine dehydrogenase from *Saccharomyces cerevisiae*, also the alanine dehydrogenase from *Phormidium lapideum* undergoes a conformational change upon substrate binding which is a perquisite for its activity [26].

In summary, substrates of OcDH bind in an ordered sequential manner. First NADH binds to OcDH followed by L-arginine. The binding of the guanidinium headgroup of L-arginine induces a conformational change, resulting in the formation of the pyruvate binding site. This implies that the reduction of pyruvate can only occur in the presence of L-arginine, which than forms octopine and prevents lactate formation as earlier already observed [27]. Also the structure of the OcDH/NADH-agmatine complex indicates that the binding of the L-arginine side chain triggers the domain closure of OcDH and not the carboxyl moiety of the amino acids, a prerequisite for catalyzing octopine synthesis by OcDH.
